# No significant difference in skin contamination during anterior cruciate ligament reconstruction with and without preoperative skin cleaning

**DOI:** 10.1002/ksa.12476

**Published:** 2024-10-03

**Authors:** Benjamin Bartek, Alexandra Völkner, Stephan Oehme, Stephen Fahy, Tobias Winkler, Tobias Jung

**Affiliations:** ^1^ Charité – Universitätsmedizin Berlin, Corporate Member of Freie Universität Berlin and Humboldt‐Universität zu Berlin, Center for Musculoskeletal Surgery Berlin Germany; ^2^ Berlin Institute of Health at Charité – Universitätsmedizin Berlin, Julius Wolff Institute Berlin Germany; ^3^ Berlin Institute of Health Center for Regenerative Therapies, Berlin Institute of Health at Charité – Universitätsmedizin Berlin Berlin Germany

**Keywords:** ACL reconstruction, antiseptic wash lotion, contamination, prophylaxis, surgical site infection

## Abstract

**Purpose:**

This prospective study aimed to assess whether preoperative antiseptic skin cleansing reduces bacterial contamination and surgical site infections (SSI) following anterior cruciate ligament reconstruction (ACLR). We hypothesized that antiseptic cleaning would lower bacterial load, reducing contamination and early infections.

**Methods:**

One hundred and nineteen patients scheduled for ACLR were included in this prospective, nonrandomized study. Individuals were divided into two groups. Patients in the intervention group applied octenisan® wash lotion daily for three days before surgery and used the wash solution instead of their usual shower gel. Additionally, they swiped their leg with octenisan® soaked gloves on the morning of the operation. The control group followed their usual wash routine with no specific instructions. Fluid samples were taken before surgery from the irrigation bag and at 15‐min intervals from the reservoir of the sterile surgical drape during the procedure. Suture material used for the ACL graft and meniscus repair were also collected for testing. The samples were subjected to a 14‐day incubation period. Follow‐up included outpatient visits at 6 weeks, 12 weeks and 6 months with a final evaluation at 12 months.

**Results:**

Contamination rates showed no significant difference between the control and intervention groups. The mean contamination rate in the control group was 6.4% (*n* = 22) and 6.6% (*n* = 24) in the intervention group (*p* = 0.28). At 12‐month follow‐up, 110 out of 119 participants were included (52 control, 58 intervention). *T* tests for age (*p* = 0.19), BMI (*p* = 0.66), and surgery duration (*p* = 0.38) showed no significant differences. No early SSI were observed in either group postoperatively.

**Conclusion:**

Our results indicate that the use of antiseptic wash lotion and gloves does not influence the risk of bacterial contamination during surgery.

**Level of Evidence:**

Level III.

**Abbreviations**
ACL: anterior cruciate ligament
ACLR: anterior cruciate ligament reconstruction
BMI: body mass index
CI: confidence interval
CNS: coagulase‐negative Staphylococci
h: hour
min: minutes
mL: millilitre
MRI: magnetic resonance imaging
*n*
: number
PJI: periprosthetic joint infection
SSI: surgical site infection

## INTRODUCTION

A rare but serious complication of arthroscopic surgery is postoperative infection, with a reported incidence of less than 1% [1, 21, 28]. When considering various arthroscopic procedures, ACL reconstruction exhibits a higher incidence of postoperative infection (ranging from 0.3% to 1.7%) compared to procedures such as meniscus surgery (with an incidence ranging from 0.2% to 0.8%) [4, 8]. Both intrinsic patient‐related risk factors such as obesity, tobacco use, prolonged corticosteroid use, male gender, and comorbidities such as diabetes, as well as extrinsic factors such as institutional surgical volume and surgical duration increase the risk of postoperative infection [5, 8, 10, 29, 30]. Previous work in our institution has found a significant correlation between surgery duration and the presence of positive microbiological findings in the samples of irrigation fluid from the reservoir on the sterile drapes during arthroscopic ACLR and meniscus repair.

High bacterial contamination rates, specifically in operations exceeding 70 min, were observed alongside low postoperative infection rates. Possible explanations include effective perioperative antibiotic prophylaxis, pathogen elimination during arthroscopic surgery via continuous joint flush, and undiscovered patient immune capacities [3, 17].

Postoperative infections are often caused by coagulase‐negative Staphylococci (CNS), *Staphylococcus aureus*, and *Cutibacterium acnes* organisms that are part of the physiological skin flora [6, 18]. Intraoperative interventions such as single‐dose antibiotic prophylaxis and routine soaking of grafts in vancomycin have been shown to significantly lower infection rates [7, 11].

Other suggested measures for reducing contamination and infection rates in orthopaedic surgery include the frequent changing of gloves and suction tips, as well as the use of laminar air flow systems [2, 12, 22].

In the last years preoperative antiseptic skin cleaning procedures have attracted interest due to their published potential to decrease SSI. Since this concept has up to date only been investigated in arthroplasty patients, we intended to analyse the effect of preoperative preparation with antiseptic wash lotion in a patient cohort undergoing ACLR. We hypothesized that preoperative antiseptic cleaning would diminish bacterial load, thereby decreasing the rate of intraoperative contamination and early postoperative infections, a concept initially posited by McAllister et al. [19].

In case of being efficacious, preoperative antiseptic measures could represent a simple yet potent addition to the arsenal against postoperative infections in ACLR patients.

## MATERIALS AND METHODS

### Study design

Ethical approval for this prospective, single‐blinded study was granted by the Charité Universitätsmedizin Berlin's ethics committee (EA1/168/21), and the study was listed with the German Clinical Trials Register (DRKS00023488). The inclusion criteria were (i) patients over the age of 18, (ii) MRI‐proven ACL‐injury, with or without associated meniscal pathology, undergoing ACLR with or without additional meniscus repair. The exclusion criteria were (i) prior knee surgery (ii) prior infection of the injured knee and (iii) patients deemed incapable of providing informed consent.

### Participants

One hundred and nineteen patients scheduled for ACLR were enroled in the study, with 60 undergoing additional meniscus repair. These individuals were divided into two groups: the intervention group (*n* = 60) used octenisan® wash lotion for 3 days before surgery and, additionally to that, octenisan® soaked gloves on the day of surgery for daily body care. The control group (*n* = 59) used their own, regular body care productes without antiseptic ingredients.

For the allocation of groups (intervention or control group), the date of the surgical preparation was used. If this was scheduled at least 3 days before the surgery, patients were offered the opportunity to use octenisan® as described before. With shorter lead times, automatic allocation to the control group occurred if patients consented to participation.

The surgical and microbiological teams were unaware of the group allocations, but it was not possible to keep the participants unaware due to the nature of the intervention.

### Intervention protocol

The antiseptic protocol involved the use of octenisan® wash lotion and octenisan® soaked washing gloves. Patients in the intervention group were instructed to use the lotion for their daily, morning and evening, body care routine 3 days before surgery. Patients were asked to use octenisan® wash solution instead of their usual shower gel for the whole body. In addition, patients of the intervention group swiped their leg with octenisan® soaked gloves on the morning of the operation. The octenisan® products were dispensed to patients either during the preoperative consent process or via postal delivery.

Patients in the control group used their regular wash lotion with no special advice.

### Surgical procedure

Three high‐volume ACLR surgeons in an academic tertiary care centre performed the surgeries. The surgeons wore standard sterile surgical gown and two pairs of surgical gloves. Patients received single‐shot perioperative antibiotic prophylaxis (Cefazolin 2 g intravenously 30 min before skin incision). In the event of cephalosporin intolerance, 1 g of vancomycin was administered intravenously 2 h before skin incision. All patients underwent shaving of the surgical site with a disposable razor immediately before surgery. Standard precleaning of the patient's lower extremity was performed using an alcoholic solution (Softasept N, B. Braun) before final disinfection. Following this, the skin was disinfected four times with a povidone–iodine solution (Braunol, B. Braun) for at least 10 min in total. Sterile draping was performed following a routine protocol, with a two‐layer sterile covering drape including one reservoir. Hamstring autografts were used for all ACLRs, with meticulous preparation. The graft was wraped with a vancomycin‐soaked compress for 60 s before implantation. When concomitant meniscal injuries were identified intraoperatively they were treated with either meniscal suturing or resection. Purisole® solution (5000 mL; Fresenius Kabi) was used as the irrigation solution. A soft wound drain (BLAKE Silicone Drain®, Ethicon) was used in all patients. No postoperative antibiotics were given in any of the evaluated cases.

### Sample collection and analysis

Before each surgery, one fluid sample was taken under sterile conditions using a syringe with cannula directly from the irrigation bag to exclude preexisting fluid contamination. After defined time intervals of 15 min each, 5 mL of irrigation fluid was collected from the reservoir of the sterile surgical drape using new syringes at all time points and injected into sterile tubes. The reservoir of the drape was not emptied during the surgery. In addition, during surgery, all suture and fixation material previously used for meniscus repair and ACL graft preparation (2–0, nonabsorbable, UHMW polyethylene ULTRABRAID™ Suture [Fast‐Fix 360; Smith and Nephew]; 2–0 and 0, absorbable, polydioxanon suture [Ethicon]) or tendon preparation (nonabsorbable polyester fibers [PremiCron®, B. Braun]) was collected in sterile tubes immediately after use for subsequent microbiological testing. The tendon preparation took place on a separate table with sterile covering.

All samples were sent to the microbiology institute, where they were incubated for 14 days. In case of positive microbiological results, bacterial species were identified and resistograms established. This experimental design was more susceptible to false‐negative than to false‐positive results as all fluid samples were taken from a relatively big reservoir with high chances of missing the overall small bacterial load and its uneven distribution. Therefore, to prevent the underestimation of contamination, cases, in which specimen from one and the same patient showed negative microbiological results after a previous culture‐positive microbiological finding, were rated as false negative.

### Follow‐up

All patients were seen in the outpatient department 6 and 12 weeks postoperatively and examined for clinical signs of surgical site infection. Six months after surgery, a telephone consultation was performed inviting each patient to join final follow‐up evaluation at our outpatient clinic. If the patient failed to appear personally, a questionnaire‐based telephone interview was conducted to inquire patient satisfaction with surgery and to evaluate knee function and condition, also evaluating the possibility of a possible infection.

### Statistical analysis

A power analysis was conducted using G*Power software, informed by previous research from our institution and a meta‐analysis reporting an odds ratio of 0.27 for preoperative antiseptic washing (11). Due to the low incidence of postoperative infections after ACL reconstruction, the study focused on detecting differences in positive microbiological findings. To achieve an 80% power and a 5% significance level, a sample size of 120 patients (60 per group) was calculated. This sample size was sufficient to detect a meaningful effect without being unnecessarily large. Regression analysis was performed using IBM® SPSS®, with patient demographics (age, gender, BMI) and surgical characteristics included as covariates.

## RESULTS

### Patient population

One hundred and nineteen patients were enroled in the study, 38 (32%) were women and 81 (68%) were men. The mean age of participants was 32.9 ± 9.8 years. The mean body mass index (BMI) was 25.3 ± 3.8 kg/m^2^. Thirty‐eight patients (32%) reported daily tobacco use (Table 1).

**Table 1:** Patient and surgery characteristics.

Variables	Control group	Intervention group	*p* Value
Age in years	31.6 ± 9.6	34.2 ± 9.9	0.12
BMI	25.2 ± 3.7	25.5 ± 4.0	0.64
Female/male in % (*n*)	30.5 (18)	33.3 (20)	0.74
Smokers in % (*n*)	40.7 (24)	23.3 (14)	0.04*
Surgery duration in minutes	61.4 ± 18.8	59.3 ± 22.9	0.54
Meniscus suture in % (*n*)	61 (36)	40 (24)	0.02*

* Statistically significant.

**Figure 1:**
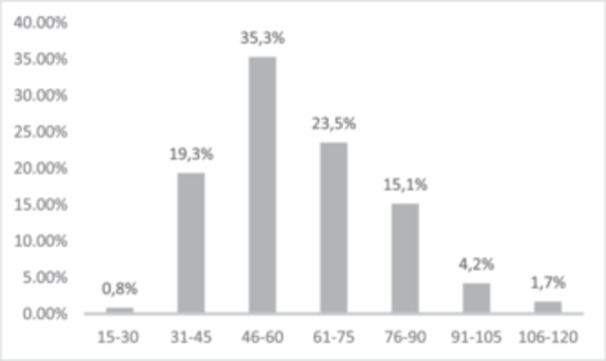
Number of ACLR in specific time frames (in min). ACLR, anterior cruciate ligament reconstruction.

The average duration for the ACLR was 51.6 ± 12.8 min and for ACLR combined with meniscus repair 68.7 ± 23.8 min (Figure 1).

The standard antibiotic prophylaxis consisted of Cefazolin. In case of intolerance, Vancomycin was administered. This scenario occurred in two patients in the intervention group and one patient in the control group.

### Contamination rate

A total of 710 samples were included in the final analysis, consisting of 345 samples from patients in the control group and 365 samples from patients in the intervention group. The latter group had a contamination rate of 6.6% (*n* = 24), the control group a rate of 6.4% (*n* = 22). The regression analysis did not reveal a statistically significant between‐group difference, with a *p* value of 0.28 from the omnibus test. The explanatory power of the model was limited, as indicated by the Cox and Snell *R* ^2^ value of 0.052 and the Nagelkerke *R* ^2^ value of 0.0075.

**Figure 2:**
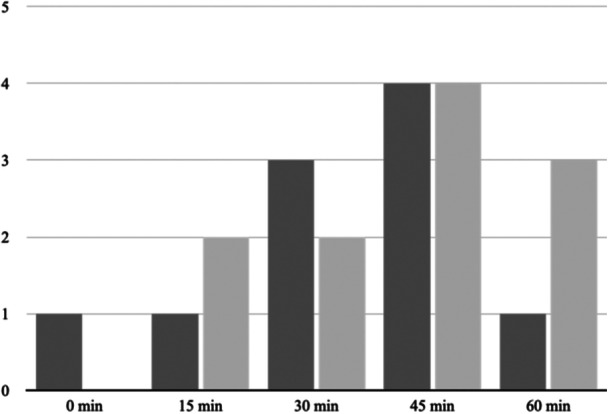
Number of contaminated samples in the fluid. Dark: samples from the control group (not washed). Light: samples from the intervention group (washed).

Contamination was found in 21 (4.8%) of the 439 arthroscopic fluid samples. Out of the 21 contaminated samples, 11 positive samples were observed in the intervention group. A positive correlation was observed between surgical duration and the risk of bacterial contamination (Figure 2).

Suture samples accounted for 38% (*n* = 271) of the investigated material. Bacterial contamination was detected in 9.3% (*n* = 25) of sutures analyzed. Twelve samples from the control group (9.1%) and 13 (9.4%) samples from the intervention group were positive for bacterial contamination.

### Pathogens

CNS were the most frequently identified pathogens, accounting for 32 (57.2%) of the contaminants. *C. acnes* were found in 8 (14.3%) of cases, and *S. aureus* in another 4 (7.2%) (Table 2).

**Table 2:** Pathogen spectrum in total and individual groups in % (*N*).

	Total	Control	Intervention	*p* Value
*Staphylococus epidermidis*	32.1 (18)	29.6 (8)	34.5 (10)	0.72
*Staphylococcus caprae*	10.7 (6)	‐	20.7 (6)	0.02*
*Staphylococcus aureus*	7.1 (4)	11.1 (3)	3.5 (1)	0.29
*Cutibacterium acnes*	14.3 (8)	14.8 (4)	13.8 (4)	0.94
*Staphylococcus capitis*	5.4 (3)	3.7 (1)	6.9 (2)	0.6
*Bacillus cereus*	3.6 (2)	7.4 (2)	‐	0.15
*Staphylococcus haemolyticus*	3.6 (2)	3.7 (1)	3.5 (1)	0.97
Other	23.2 (13)	29.6 (8)	17.3 (5)	0.35

* Statistically significant.

### Follow‐up

Over the follow‐up period, 114 patients attended the 6‐week check‐up and 110 returned for the 3‐month visit. Importantly, within 3 months after ACLR, none of the patients in the study developed early postoperative joint infections. We could reach 117 patients for the 6‐month telephone interview and 110 patients for the 12‐month interview. None of these patients reported any signs of surgical site infection.

## DISCUSSION

The most important findings of our study demonstrate that preoperative antiseptic washing, although expected to reduce bacterial load and intraoperative contamination during ACLR, did not lead to a significant reduction in intraoperative bacterial contamination or postoperative surgical site infection rates. Despite the anticipated benefits of using octenisan® wash lotion to decrease skin flora microorganisms, our results showed no notable difference in these outcomes with the implementation of antiseptic washing measures.

The literature regarding the efficacy of preoperative skin cleaning in orthopaedics is limited, with no current consensus and guidelines. Many of the proponents of preoperative skin cleaning cite the landmark research by Rotter and colleagues in 1987 as clear evidence of benefit. The authors found an approximately 30% reduction in surgical site infection rates across a variety of surgeries, with a 50% decrease in rates of *S. aureus* wound contamination in ‘clean’ surgeries through the use of chlorhexidine wash lotion [27]. Studies focusing on orthopaedic surgery are rare, but often show that antiseptic precleaning has minimal to no impact on postoperative surgical site infection rates when compared with regular soap or routine preoperative preparation [13, 14]. The findings of this study have clinical implications, particularly in the context of ACLR. Our results suggest that the routine use of preoperative antiseptic wash lotions, such as Octenisan®, may not be as beneficial as previously assumed in reducing intraoperative contamination and subsequent infection rates. This is particularly important considering the time, cost, and patient compliance required for such preoperative interventions. By questioning the efficacy of these commonly recommended antiseptic practices, our study highlights the need for a more targeted approach to infection prevention that considers the specific microbiota involved and the timing of antiseptic application. Ultimately, this could lead to more efficient and cost‐effective surgical protocols, minimizing unnecessary steps in the preoperative phase without compromising patient safety.

The contamination rates observed in both irrigation fluid and suture materials within our study are comparable with those reported in previous studies, although a direct comparison is challenging due to the heterogenous nature of the surgical procedures [12, 14, 15]. The level of bacterial contamination is comparable with rates seen in arthroscopic shoulder surgery suggesting that the surgical site may have a limited impact on infection risk and that other factors exist in both the preoperative and intraoperative phase which may be contributing to bacterial contamination on suture materials [14, 15].

Our findings are consistent with recent studies highlighting the vulnerability of hamstring tendon autografts to contamination during ACLR. Offerhaus and colleagues reported a notable incidence of bacterial contamination, predominantly involving skin flora such as *Staphylococcus epidermidis* and *Staphylococcus capitis*, with 42.4% of patients exhibiting bacterial presence in harvested tendons [24]. These results support the hypothesis that bacterial contamination primarily occurs during the graft harvesting and preparation stages. In our study, contamination rates similarly reflected the frequent presence of skin commensals, with *S. epidermidis* being commonly isolated.

Furthermore, literature have shown that contamination does not always lead to clinical infection [26]. For instance, despite the high contamination rates reported by Offerhaus and colleagues, no deep knee infections were observed within the 1‐year follow‐up period [24]. This suggests that effective intraoperative and postoperative prophylactic measures, such as the use of vancomycin‐soaked grafts, can significantly mitigate the risk of surgical site infections, even in the presence of contamination. The consistent susceptibility of isolated bacteria to vancomycin further supports the routine use of this antibiotic in ACLR procedures to reduce postoperative infection rates [26].

We frequently detected CNS in both the control and intervention groups, consistent with the established prevalence of CNS as contaminants in ACLR. Pérez‐Prieto and colleagues isolated similar bacterial strains from ACL graft samples while Offerhaus and colleagues identified CNS in as causative agent in the majority of ACLR‐related surgical site infections [23, 25]. Octenisan® has broad‐spectrum antimicrobial activity, encompassing both Gram‐positive and Gram‐negative bacteria, as well as fungi. However, the specific pathogens predominantly identified in our study, namely CNS and *C. acnes*, are recognized for their persistence on the skin despite standard antiseptic protocols. While Octenisan® has shown efficacy in reducing bacterial load, its effectiveness may vary depending on the skin microbiota and the surgical context [16]. The literature indicates that chlorhexidine‐based antiseptics, such as chlorhexidine gluconate, have been more extensively investigated and are frequently recommended in surgical environments due to their prolonged antimicrobial activity and established effectiveness in reducing surgical site infection (SSI) rates, particularly against *S. aureus* [16]. Considering the prevalence of CNS and other skin flora organisms in our findings, it could be beneficial to examine whether a chlorhexidine‐based antiseptic might produce different outcomes in a similar setting. This consideration is particularly relevant for procedures such as ACLR, where the skin microbiota may contribute to the risk of contamination [20]. Additionally, the timing of antiseptic application relative to the surgical procedure could influence its effectiveness. In our study, patients in the intervention group applied Octenisan® wash lotion for three consecutive days prior to surgery, with an additional application on the morning of the surgery. The interval between the final application and the time of surgery may have impacted the persistence of the antiseptic effect. The antimicrobial efficacy of topical antiseptics can diminish over time, depending on the agent used, the microbial load and the characteristics of the skin. For instance, chlorhexidine‐based antiseptics are known for their prolonged activity, whereas the duration of the antimicrobial effect of octenidine dihydrochloride may be shorter. Given this context, the interval between the last application of Octenisan® and the time of surgery might have allowed for some re‐colonization of the skin by resident flora [9].

Despite the efforts for a rigorous methodology, there are limitations to this study. Our study is focused on contamination rates rather than SSI, which limits our ability to draw definitive conclusions about the effectiveness of preoperative antiseptic measures in preventing SSI. Additionally, while our sample size was calculated to detect differences in contamination rates, the study was not powered to assess the impact on the actual infection rates due to the low incidence of postoperative SSI following ACLR. This statistical limitation suggests that larger studies are needed to fully understand the role of preoperative antiseptic practices in reducing SSI risks.

The lack of randomization is a potential source of bias that could affect the validity of our results. However, to address this concern, we conducted statistical analyses to compare key variables between the intervention and control groups. Group allocation was determined based on the timing of surgical preparation, without any additional selection criteria. The analyses demonstrated that the groups were generally well‐matched across most variables, with no significant differences in age, BMI, operation duration or gender. Although a significant difference was found in smoking status, the overall distribution of key variables helps mitigate the disadvantage of the missing randomization.

We endeavored to minimize bias by restricting the number of surgeons involved, implementing standardized perioperative management and surgical procedures and ensuring that study staff remained unaware to the assigned groups.

Furthermore, the relatively short follow‐up period of 12 months may have not allowed to detect all, particularly low‐grade infections. Therefore, the true rate of joint infection could be higher. However, a systematic review and meta‐analysis by Di Zhao and colleagues investigated the time from surgery to the onset of surgical site infection symptoms. The pooled analysis showed that the mean time from surgery to the onset of surgical site infection symptoms was 17.1 days (95% CI, 13.2–21.0 days; *I* ^2^ = 77%) [30].

## CONCLUSION

In conclusion, this study challenges the assumed benefits of routine preoperative antiseptic wash lotions, such as Octenisan®, in the context of ACLR, suggesting they may not significantly reduce intraoperative contamination.

## AUTHOR CONTRIBUTIONS

**Benjamin Bartek**: Conceptualization; methodology; writing—original draft preparation; writing—review and editing; supervision. **Tobias Jung**: Conceptualization; methodology; supervision. **Tobias Winkler**: Methodology; writing—review and editing; supervision. **Alexandra Völkner**: Data curation. **Stephen Fahy**: Writing—review and editing. **Stephan Oehme**: Writing—review and editing. All authors have read and agreed to the published version of the manuscript.

## CONFLICT OF INTEREST STATEMENT

The authors declare no conflict of interest.

## ETHICS STATEMENT

The study was approved by the institutional review board of the Charité, Universitätsmedizin Berlin (EA1/168/21), and the study was listed with the German Clinical Trials Register (DRKS00023488). Informed consent was obtained from all individual participants included in the study.

## Data Availability

The authors confirm that the data supporting the findings of this study are available within the article and its Supporting Information. Raw data that support the findings of this study are available from the corresponding author, upon reasonable request.
